# Current trends in tendinopathy: consensus of the ESSKA basic science committee. Part II: treatment options

**DOI:** 10.1186/s40634-018-0145-5

**Published:** 2018-09-24

**Authors:** F. Abat, H. Alfredson, M. Cucchiarini, H. Madry, A. Marmotti, C. Mouton, J. M. Oliveira, H. Pereira, G. M. Peretti, C. Spang, J. Stephen, C. J. A. van Bergen, L. de Girolamo

**Affiliations:** 1 Department of Sports Orthopaedics, ReSport Clinic, Passeig Fabra i Puig 47, 08030 Barcelona, Spain; 2 Sports Medicine Unit, University of Umeå, Umeå, Sweden; 3 Alfredson Tendon Clinic Inc, Umeå, Sweden; 4 Pure Sports Medicine Clinic, ISEH, UCLH, London, UK; 5 Molecular Biology, Center of Experimental Orthopaedics, Saarland University Medical Center, Kirrbergerstr. Bldg 37, D-66421 Homburg, Saar Germany; 6 Lehrstuhl für Experimentelle Orthopädie und Arthroseforschung, Universität des Saarlandes, Gebäude 37, Kirrbergerstr. 1, D-66421 Homburg, Germany; 7 Department of Orthopaedics and Traumatology, San Luigi Gonzaga Hospital, Orbassano,University of Turin, Turin, Italy; 8 Department of Orthopedic Surgery, Clinique d’Eich-Centre Hospitalier de Luxembourg, 76, rue d’Eich, L-1460 Luxembourg, Luxembourg; 9 3B’s Research Group – Biomaterials, Biodegradables and Biomimetics, University of Minho, Headquarters of the European Institute of Excellence on Tissue Engineering and Regenerative Medicine, AvePark, Zona Industrial da Gandra, 4805-017 Barco GMR, Portugal; 10 ICVS/3B’s - PT Government Associate Laboratory, Braga, Guimarães Portugal; 11 The Discoveries Centre for Regenerative and Precision Medicine, Headquarters at University of Minho, Avepark, 4805-017 Barco, Guimarães Portugal; 12 Orthopedic Department Centro Hospitalar Póvoa de Varzim, Vila do Conde, Portugal; 13 Ripoll y De Prado Sports Clinic – FIFA Medical Centre of Excellence, Murcia, Madrid Spain; 14 IRCCS Istituto Ortopedico Galeazzi, Department of Biomedical Sciences for Health, University of Milan, Milan, Italy; 15 Department of Integrative Medical Biology, Anatomy Section, Umeå University, Umeå, Sweden; 16 Fortius Clinic, 17 Fitzhardinge St, London, W1H 6EQ UK; 17 The Biomechanics Group, Department of Mechanical Engineering, Imperial College London, London, UK; 18 Department of Orthopedic Surgery, Amphia Hospital Breda, Breda, The Netherlands; 19 Orthopaedic Biotechnology Laboratory, Orthopaedic Institute Galeazzi, Milan, Italy

## Abstract

The treatment of painful chronic tendinopathy is challenging. Multiple non-invasive and tendon-invasive methods are used. When traditional non-invasive treatments fail, the injections of platelet-rich plasma autologous blood or cortisone have become increasingly favored. However, there is little scientific evidence from human studies supporting injection treatment. As the last resort, intra- or peritendinous open or endoscopic surgery are employed even though these also show varying results. This ESSKA basic science committee current concepts review follows the first part on the biology, biomechanics and anatomy of tendinopathies, to provide a comprehensive overview of the latest treatment options for tendinopathy as reported in the literature.

## Introduction

The great incidence of tendon injuries in the population as well as the failure rate of up to 25% (Lohrer et al. 2016) of the available conservative treatments has made this field one of the most interesting for alternative biological approaches. The study of the microenvironment of tendinopathy is a key factor in improving tendon healing. There is still debate around the true role of inflammation and of overload in the activation of the processes. They are both factors that gradually produce degenerative changes of the tendon structure due to qualitative and quantitative alterations of tenocytes (Abate et al. 2009). Historically, tendinopathy has primarily been considered a degenerative pathological process of a non-inflammatory nature as the presence of acute inflammatory cells in chronic tendinopathy has never been confirmed. However, thanks to the newer research tools, convincing evidence that includes an increasing number of inflammatory cells in pathological tendons (Dean et al. 2016) has started to appear showing that the inflammatory response is a key component of chronic tendinopathy (Rees et al. 2014). For example, an increase in terms of cytokines, inflammatory prostaglandins, and metalloproteinases (MMPs) along with tendon cell apoptosis seem to be provoked by continuing mechanical stimuli (Andres and Murrell, 2008; Rodriguez et al. 2015). In this context, an alternative anti-inflammatory and immunomodulatory approach that replaces the traditional anti-inflammatory modalities (i.e. NSAIDs) may provide another potential opportunity in the treatment of chronic tendinopathies. In a previous report, biology, biomechanics, anatomy and an exercise-based approach were discussed (Abat et al. 2017). The current concepts review here provides an overview of the some treatment options for tendinopathy as reported in the literature.

## Treatment options

### Platelet RICH plasma (PRP)

The use of Platelet Rich Plasma (PRP) for the treatment of tendinopathy is a greatly debated topic in literature. The common perception that it “may” be useful in clinical settings has led to the wide spread use of PRP to treat acute and chronic tendon injuries in both Europe and the United States although conflicting evidence still exists as to its efficacy and the form in which PRP should be used.

A recent systematic review (Filardo et al. 2016) has highlighted the controversial results of PRP applications for different pathologies. The authors affirm that, following the current evidence, patellar and lateral elbow tendinopathy showed improvement from PRP treatment while the Achilles tendon and rotator cuff do seem not to benefit from PRP application with either conservative treatment or surgery. Conversely, the recent meta-analysis by Fitzpatrick (Fitzpatrick et al. 2017) has shown good clinical evidence that favors the use leukocyte-rich PRP (LR-PRP) under ultrasound guidance for the treatment of patellar tendinopathy, lateral epicondylitis and Achilles and rotator cuff tendinopathy. Similarly, the study by Pandey (Pandey et al. 2016) showed a positive result from the application of a moderately concentrated leukocyte-poor PRP (LP-PRP) above the repair site during single-row arthroscopic repair of large degenerative cuff tears. On the other hand, a prior study by Zumstein (Zumstein et al. 2016) failed to show any benefit from the application of PRP in the form of a leucocyte and platelet-rich fibrin matrix during arthroscopic rotator cuff repair.

The fact is that there is no consensus. This is mainly due to the lack of standard PRP preparation procedures or methods of application. This, at present, suggests caution in the indiscriminate first-line application of PRP in tendon disorders. Nevertheless, basic science studies may be the key to bringing the biological rationale for PRP into safe clinical usage. Indeed, the most recent in vitro and preclinical studies have shown some important clues as to the action of PRP and the proper composition to be used on tendon cells. Even if it seems that animal derived PRP has less favorable properties than human PRP, as has been observed in different settings like that of bone formation (Plachokova et al. 2009), preclinical observations may give well-defined evidence of the mechanism of PRP.

Firstly, the in-vitro study by Hudgens (Hudgens et al. 2016) with rat fibroblasts has demonstrated that one of the early responses to PRP application in rats is intermittent bouts of inflammation. They used a manually prepared PRP with leukocytes and a 4-fold elevation in the platelet concentration. Similarly to cartilage-like tissue, in which the connection between a transient early inflammatory process and the expression of inflammation related NF-ĸB subunit p65 and chondrogenic differentiation (Caron et al. 2012; Caron et al. 2014), Hudgens (Hudgens et al. 2016) has observed the activation of pro-inflammatory Tumor Necrosis Factor TNF-alpha and NFkB pathways after PRP exposure as well as the expression of genes related to cellular proliferation and tendon collagen remodeling. This explains an initial transient inflammatory response to PRP that may be more pronounced if it is in the presence of leukocytes. In chronic tendinopathies, inducing an acute bout of inflammation may represent a key element in triggering a subsequent regenerative response. It may also partially sustain the positive result of PRP in chronic tendon degeneration.

The control of the inflammatory process by PRP seems to derive from a key element in PRP, namely the hepatocyte growth factor (HGF). HGF is not simply a trophic factor and an anti-fibrotic regulator. It has been previously recognized as the main factor responsible for the PRP anti-inflammatory effect on human chondrocytes through inhibition of NF-kB transactivating activity (Bendinelli et al. 2010). A recent study by Zhang (Zhang et al. 2013) has suggested a similar effect in rabbit and mouse LR-PRP with a four-fold platelets concentration than in whole animal blood in an in vitro rabbit tenocytes culture and in a preclinical mouse model of an acute Achilles tendon lesion. It is likely that HGF is not only delivered by the platelets but also produced by cells following exposure to PRP. This presence of HGF may partially explain the secondary reduction of the first initial PRP-induced inflammatory phase.

A parallel and transient increased expression of transforming growth factor (TGF)-beta following PRP exposure has been recently described by Lyras (Lyras et al. 2010) as a key factor in accelerating tendon healing. The authors set up a patellar tendon defect model in rabbits treated with intralesional PRP gel. TGF-beta has been shown to increase during the first 2 weeks, consistent with an anabolic stimulus, and then decrease after this initial phase. That likely leads to a reduction in adhesion and scar formation. The same authors described this PRP-driven angiogenesis during tendon healing (Lyras et al. 2010) as likely being driven by the expression of vascular endothelial growth factor (VEGF) and other angiogenetic factors. PRP has been shown to temporally increase the angiogenetic phase and subsequently lead to a prompt reduction of this phenomenon, thus accelerating the whole tendon healing process.

These different anabolic mechanisms of PRP seem to be impaired by the presence of leukocytes. Indeed, the recent works of Fortier and co-workers (Boswell et al. 2014; Cross et al. 2015; McCarrel et al. 2012) have shown that a high white blood cell concentration leads to a predominant expression of inflammatory and degradative factors like Interleukine (IL-1) beta and MMP-9, while LP-PRP was related to a greater content in IL-6 that is associated with anti-inflammatory and regenerative effects in the healing tendon. This is in accord with the recent study by the Andia’s group (Rubio-Azpeitia et al. 2016). It demonstrates enhanced COL1A1, COL3A1, decorin, fibronectin, aggrecan and connective tissue growth factor (CTGF) expression and reduced MMP-1 expression after exposure to LP-PRP. Similarly, the recent in vitro study by Zhang (Zhang et al. 2016) showed that the exposure of rabbit tendon stem cells to LR-PRP decreased expression of VEGF, epidermal growth factor (EGF), Transforming Growth Factor Beta − 1 (TGF-β1) and platelet-derived growth factor (PDGF). Moreover, it reduced the production of collagen when compared to LP-PRP.

However, there may still be a role for leukocytes in PRP. A recent rabbit study by Zhou (Zhou et al. 2015) suggests that a PRP preparation with a small number of leukocytes may be beneficial in treating acute tendon lesions and early stage healing. In these situations, the marked anabolic effects of PRP without white blood cells may induce an excessive amount of collagen and matrix production that likely leads to scar formation. Conversely, the presence of a small number of white blood cells may counterbalance this anabolic effect and lead to controlled inflammation and a more physiological tendon healing (Pandey et al. 2016). It has been suggested that the use of PRP with a moderate leukocyte concentration improves arthroscopic repair of rotator cuff tears. On the other hand, Zhou et al. (Zhou et al. 2015) suggested that high levels of leukocytes are harmful in any case because they have been seen to induce a catabolic environment as well as predominant collagen type III production that may lead to scar formation and impaired tendon healing. Moreover, the authors recommend the use of a PRP without leukocytes in treating already-inflamed tendinopathic tendons and in late stage healing. This is the case in which the anabolic actions and low inflammatory effects of PRP should be prevalent and the persistence of the inflammatory phase driven by the leukocytes, conversely, would impair the healing process.

In addition, a recent work has introduced a new perspective on the use of leukocytes (Yoshida and Murray 2013). They observed a significant improvement in collagen production by fibroblasts using a neutrophil depleted-monocyte enriched PRP. This new solution would make it possible to preserve the anabolic properties of monocytes while reducing the catabolic effect of cytokines produced by neutrophils. Although it is a very interesting perspective, only future studies will clarify the possible clinical relevance of this approach.

Furthermore, two different pieces of evidence were also laid out in very recent in-vitro observations.

First, the concept of “the more, the better” does not seem to be beneficial during the preparation of PRP for tendon disease (Boswell et al. 2014). Indeed, increasing the number of platelets over 1x10E6/ul may paradoxically reduce cell proliferation (Giusti et al. 2014).

Second, a remarkably reduced response to PRP was observed in degenerated tendons. In terms of the in-vitro setting of the “diseases-in-a-dish”, both Cross (Cross et al. 2015) and Zhang (Zhang and Wang 2014) have described a lack of response to PRP in explants or tendon stem cells from late-stage tendinopathy. This may partially explain some of the conflicting data coming from trials and meta-analyses and it may justify the in vivo application of PRP in a clinical setting in which a biological response is the aim. Indeed, PRP is not able to reverse the degenerative conditions of late-stage tendinopathy in which the infiltration of mononuclear cells, permanent neovascularization, the metaplastic non-tenocyte differentiation of tendon cells and the non-tendinous tissues are predominant. In these cases, only the surgical debridement of tendon degenerated areas followed by PRP application may improve tendon quality (Zhou and Wang 2016).

All the evidence suggests that PRP may have an effective role in treating tendon pathology if careful “attention to the details” of PRP preparation goes together with a thorough knowledge of the clinical setting in which the specific PRP will be used. The “one size fit all” approach is not sustainable due to the complexity of tendon pathology and the variability of the PRP preparation steps. Indeed, not only the number of leukocytes and platelets should be known, but many other factors in the whole application process may influence the final results (Jovani-Sancho et al. 2016). Some examples are cited below.

I) The anticoagulant used during blood extraction: It is known that the common ethylene-diaminetetraacetic acid (EDTA) causes platelet inhibition and fragmentation.

II) The activation method: It is included because bovine thrombin may lead to a reduction in total growth factor concentrations and a faster release of growth factors due to the very short coagulation time. On the other hand, there is local PRP activation by means of collagen type I in a damaged tissue leads to less clot retraction than those formed with bovine thrombin.

III) The administration method: Ultrasound guidance is an emerging feature for a clinical use of PRP. It leads to better results in terms of the precision of intratendinous PRP delivery into the affected area. Indeed, recent studies by Fitzpatrick (Fitzpatrick et al., 2017), Jacobson (Jacobson et al. 2016), Dallaudière (Dallaudière et al. 2014) and Wesner (Wesner et al. 2016) have shown that ultrasound-guided intratendinous PRP injection may lead to both clinical and MRI improvements in tendon pathology.

IV) The number of injections: Some studies have shown the superiority of multiple injections of PRP (one or two weeks apart) over the single injection in different clinical settings like chronic patellar tendinopathy (Zayni et al. 2015; Charousset et al. 2014) and Achilles tendinopathy (Filardo et al. 2014).

V) The use of local anesthetics during PRP injections: A study by Bausset (Bausset et al. 2014) has shown that there is a possible detrimental effect on platelet aggregation.

VI) The rehabilitation program after PRP treatment: The biological stimulation of PRP leads to better results in combination with patient adherence to the rehabilitation protocols (Filardo et al. 2018).

So, the future of PRP for tendon pathology is still open and basic science studies continue to support its role in facilitating tendon healing, at least in the early phases. The connection between the preclinical premise and specific standardized clinical protocols for PRP preparation for acute and chronic lesions is the key element in allowing for the front-line clinical application of PRP in the treatment of tendon diseases and tendinopathy.

### Ultrasound guided galvanic electrolysis technique (USGET)

In recent years, the UltraSound-guided Galvanic Electrolysis Technique (USGET) has emerged in the scientific literature (Abat et al. 2014; Abat et al. 2015; Moreno et al. 2017), given the good results yielded in the treatment of refractory tendon injuries in comparison to other previous conservative treatments (Abat et al. 2016).

**Fig. 1:**
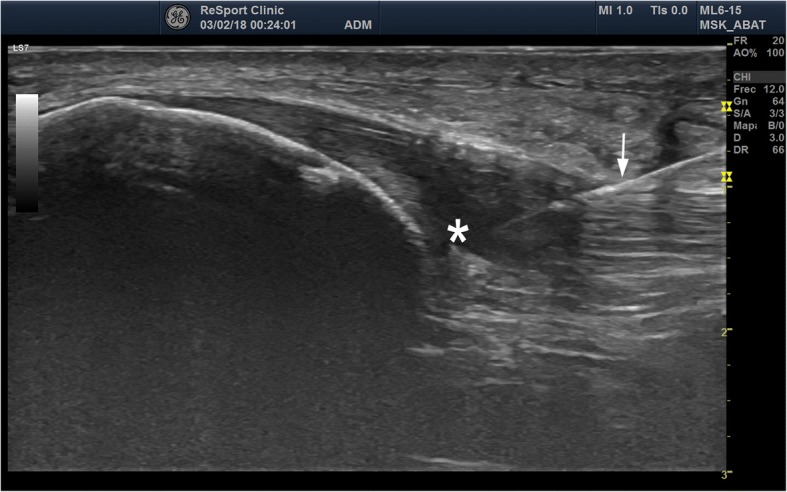
US image with 6-15MhZ linear probe showing patellar tendinopathy (*) with thickening and hipoecogenic areas. USGET through 0.3 mm needle (arrow) was applied

USGET is non-thermal electrochemical ablation with a cathodic flow to the clinical focus of tendon degeneration (Fig. 1). This treatment produces a dissociation of water, salts and amino acids in the extracellular matrix that creates new molecules through ionic instability. The organic reaction, which occurs in the tissue around the cathodic needle, causes a localized inflammation in the region dealt with (Abat et al. 2014). It produces an immediate activation of an inflammatory response and overexpression of the activated gamma receptor for peroxisome proliferation (PPAR-gamma). Furthermore, it acts to inhibit the action of IL-1, TNF and COX-2, mechanisms of tendon degeneration through the direct inhibitory action of factor NFKB that facilitates phagocytosis and tendon regeneration (Abat et al. 2014). The effectiveness of USGET in combination with eccentric exercises has been demonstrated in recent studies (Abat et al. 2015, Abat et al. 2016; Mattiussi and Moreno, 2016; Moreno et al. 2017).

The application of USGET leads to the production of new immature collagen fibers that become mature by means of eccentric stimulus (Abat et al. 2015), thereby obtaining excellent results in the short and long-term in terms of pain and function. It should be stated that the use of this techniques without the combination with mechanical stimuli results in a significant decrease in the biological effect.

The application of USGET should be limited to trained professionals and under ultrasound guidance. The application of local anesthesia is strongly recommended to avoid pain during the procedure. Nowadays, the application of USGET is indicated every 15 days so that a complete inflammatory period is fulfilled between treatments.

There are different electrolysis application methods in the existing literature. Some authors use a dosage ranging from 1 to 8 milliAmps (Abat et al. 2014) while other authors use a microAmps range (Arias-Buría et al. 2015). That fact creates big differences in the treatment intensity applied to patients and probably in the healing response, too. USGET at between 2 to 8 milliamps every 15 days is suggested. Other electrolysis techniques use a weekly dose of 2 to 4 milliamps (Moreno et al., 2017) or 350 microAmps (Arias-Buría et al. 2015) with different results.

The lack of sufficient Level 1 studies and meta-analyses makes more high-quality studies necessary to establish the indisputable efficacy of this technique.

### Mesenchymal stem cells

Although attention was mainly focused on their ability to differentiate and to directly participate to the regeneration process in the past, mesenchymal stem cells (MSCs) have more recently been demonstrated to have further and probably more important therapeutic functions in response to injury like immune modulation and trophic activities. That is why that they have been defined as “drugstores” (Caplan and Correa 2011). Indeed, they can home in on sites of inflammation or tissue injury and they start to secrete immunomodulatory and trophic agents such as cytokines and growth factors aimed to re-establish physiological homeostasis in response to that environment (Caplan and Correa 2011). So, either as direct player in the process or/and bioactive molecules “drugstores”, MSCs may enhance tissue repair and regeneration and thereby restore normal joint homeostasis. All these features, combined with the relative easy process of isolation and expansion have made MSCs potentially very useful in recent years for many clinical applications.

It is now clear that the ubiquity of MSCs is due to their origin as they derive from perivascular cells called pericytes and are thus located in all vascularized tissues. Both microvascular pericytes (Crisan et al. 2008) and adventitial cells (Corselli et al. 2012) are immunophenotipically indistinguishable from MSCs, hence the term pericytes to describe these cells. Despite their ubiquity, only specific sites have been identified as points to obtain the considerable number of cells needed for regeneration purposes (Marmotti et al. 2014). Among adult stem cells, those isolated from bone marrow (BMSCs) are the most commonly used and studied. Used alone or in association with scaffolds, BMSCs have shown to be effective for the regeneration of different tissues, including the tendon (Marmotti et al. 2014; Omi et al. 2015).

Another smart option is subcutaneous adipose tissue. It is possible to isolate MSCs, named adipose-derived mesenchymal stem cells (ASCs) from there with a simple and scarcely invasive method (Sacerdote et al. 2013). If compared to bone- and cartilage-related pathologies, the use of MSCs in tendon related disorders has been investigated very little, so far. Few animal models with different recipient and pathological conditions have been tested to date. In a study performed on surgically detached and repaired rat supraspinatus tendons, ASCs seeded on a collagen carrier were unable to increase the biomechanical parameters in comparison to the control group (Valencia Mora et al. 2014). On the other hand, a rabbit model of complete deep digital flexor tendon transection treated with suture and intratendinous injection of allogeneic uncultured rabbit ASCs showed improvements in biomechanical parameters such as stiffness and energy absorption in comparison to saline and BMSCs injected controls (Behfar et al. 2014). In a similar rabbit model, after Achilles tendon transection and suture, PRP alone or PRP with rabbit ASCs were applied to the injury site. The addition of ASCs resulted in a significant increase in tensile strength and collagen 1, VEGF and FGF production whereas TGF-beta levels diminished in comparison to using PRP alone. It confirms the effectiveness of ASCs in enhancing tissue healing but raises questions about the complex interaction of the molecular pathways induced by the treatment (Uysal et al. 2012).

Tendinopathy of the superficial digital flexor tendon was chemically induced in horses and the animals were then treated with the injection of autologous ASCs and PRP. The results showed that progression of the pathology was prevented. Additionally, there was a decrease in inflammatory infiltrate and greater organization of collagen fibers in ASC-treated tendons with respect to control animals treated with saline solution (Carvalho Ade et al. 2013). Many of the findings come from the equine clinical veterinary literature, which is often a good basis for information on the use of MSCs in humans. Especially in horses, good clinical evidence is shown for combination of PRP/MSCs (Ricco et al. 2013, Smith et al., 2013). The BMSC treated animals showed statistically significant improvements in structural stiffness, histological scoring, vascularity, water content, GAG’s content and MMP-13 activity (Smith et al. 2013). The promising data acquired from previous studies together with the lack of adverse findings support the use of this treatment option for human tendon injuries. Nevertheless, only few studies have investigated the effect of MSCs in clinical application (Wang et al. 2013; Pascual-Garrido et al. 2012; Singh, 2014; Ellera Gomes et al. 2012). A recent pilot study showed the results of the injection of allogenic ASCs mixed with fibrin glue into the common extensor tendon lesions of 12 patients with chronic lateral epicondylitis. At the one-year follow up, no significant adverse events were observed and there was a significant improvement of pain and elbow performance scores (Lee et al. 2015). The use of allogenic cells is feasible as MSCs have been demonstrated not to be very immunogenic due to the low expression of MHC class I molecules and the lack of MHC class II molecules (Prockop 2009). In another study, patients who were refractory to conservative treatment were injected with autologous BMSCs in the patellar tendon lesion. At the 5-year follow-up, a statistically significant improvement was seen for most clinical scores (Pascual-Garrido et al. 2012). In a recent randomized controlled study comparing the efficacy of adipose-derived stromal vasculat fraction and PRP, 23 patients were assigned to the PRP group and 21 to the SVF group, treated unilaterally or bilaterally for a total of 28 tendons per group. All patients (age 18-55 years) were clinically and radiologically assessed up to 6 months from the treatment. Both treatments allowed for a significant improvement with respect to baseline at the last follow-up, but comparing the two groups, the patients treated with SVF obtained faster results, with significant imporvements already after 15 days from the treatment, thus suggesting that this treatment should be taken into consideration mainly for those patients who require an earlier return to daily activities or sport (Usuelli et al. 2018). These clinical findings together with the huge amount of data derived from both in vitro and pre-clinical investigations lead to hypothesizing a role for MSCs in the treatment of tendinopathy. It is something that is also supported by the high safety profile of this procedure.

However, many issues around their application have not been completely addressed, such as the timing of MSC delivery at the injury site. Some evidence seems to suggest not delivering MSCs during the first phases of the injury process as it could result in undesired pro-inflammatory effects. Then again, doing it later may promote a desired immunosuppression process leading to injury resolution.

### Gene therapy

The concept of using gene transfer procedures to address such issues is based on the concept of providing therapeutic gene sequences that may durably enhance the healing responses and restore the original functions of the injured tendon. Based on critical advances in the understanding of tendon biology, physio- and patho-physiology as well as the mechanisms underlying tendon repair, active experimental and translational research has provided evidence of the benefits of gene therapy to address such disorders, especially by applying gene coding for diverse tenogenic factors that may promote neo-tendon formation and tendon healing over sustained periods of time relative to the injection of recombinant molecules with very short pharmacological half-life (Madry et al. 2011).

#### Principles of gene therapy

Gene therapy is the procedure to deliver gene sequences to a target cell or tissue to promote the expression of a therapeutic protein (regenerative medicine) or to correct mutated genes (monogenic disorders) (Madry et al. 2011). The administration of therapeutic sequences to treat tendinopathies has been performed using non-viral and viral (adenoviruses, retro−/lentiviruses, recombinant adeno-associated virus (rAAV) that utilize natural cellular entry pathways) gene vehicles (Table 1). Upon vector uptake at the target cell membrane, the transgene translocates towards the nucleus where it is expressed via the host cell machinery. It may then either become integrated as a part of the cellular genome or remain extrachromosomal as an episome. Enough cells need to be modified by gene transfer to permit expression of adapted levels of a transgene product.

**Table 1:** Gene therapy vectors

Class	Main advantages	Key limitations
Non-viral	not infectious, not toxic	low efficacy, short-term transgene expression
Adenoviral	high efficacy	immunogenic, short-term transgene expression
Retro−/lentiviral	long-term transgene expression	risk of insertional mutagenesis, restricted host-range, only for dividing cells (retroviral vectors), HIV-based material (lentiviral vectors)
rAAV	high efficacy, long-term transgene expression, also for quiescent cells	complex to prepare, size limitation

Non-viral vehicles (Chen et al. 2014, Goomer et al. 2000, Jayankura et al. 2003, Jiang et al. 2016, Nakamura et al. 1996, 1998, Özkan et al. 1999, Tian et al. 2015, Wang et al. 2004, 2005, Yuan et al. 2004, Zhu et al. 2006) are simple to produce and show no immunogenicity a packaging limit. However, they are less effective than viral vectors and mediate only short-term expression of the foreign material being carried because of their maintenance as unstable episomes. They are generally employed in ex vivo settings that allow for the selection of the modified cells.

Adenoviral vectors (Cai et al. 2013, Dai et al. 2003, Gerich et al. 1996, 1997, Lou et al. 1996, 2000, 2001, Majewski et al. 2008, 2012, Otabe et al. 2015, Otabe et al. 2015, Rickert et al. 2005, Schnabel et al. 2009, Zhu et al. 2006) promote very high transduction efficiencies (especially in vivo) but they are highly immunogenic while permitting only very brief levels of transgene expression due to their episomal genome (no more than 1–2 weeks).

Retro−/lentiviruses (Chen et al. 2015, Gao et al. 2016, Gerich et al. 1996, 1997, Noack et al. 2014) are integrative vectors that mediate long-term transgene expression, but they may activate the expression of tumour genes upon insertional mutagenesis. Moreover, retroviral vectors are only capable of targeting dividing cells, showing high cell specificity. On the other side, lentiviral vectors may also modify quiescent cells, but they carry deleterious sequences derived from the pathogenic human immunodeficiency virus (HIV).

Vectors based on the adeno-associated virus (AAV, a replication-defective human parvovirus) (Basile et al. 2008, Hasslund et al. 2014, Tang et al. 2008, 2014, 2016, Wang et al. 2005, 2007, Zhu et al. 2006) are safer. They exhibit low immunogenicity (no viral coding sequences are present in the recombinant genome) and mediate sustained transgene expression in a stable episomal form (months to years) in both quiescent and dividing cells, but they are relatively complex to produce and are still limited in size.

#### Gene-based approaches for tendinopathies

**Fig. 2:**
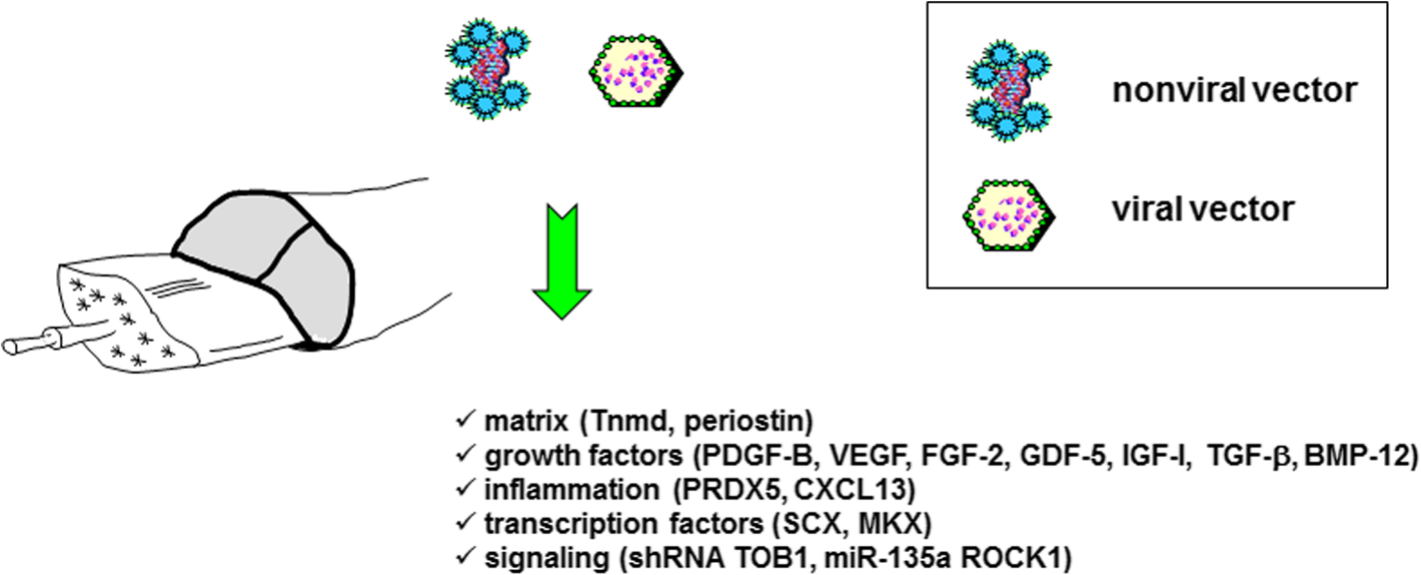
Gene transfer strategies for tendon injuries. Experimental approaches towards neotendon formation and tendon healing. Tnmd, tenomodulin; PDGF-B, platelet-derived growth factor B; VEGF, vascular endothelial growth factor; FGF-2, basic fibroblast growth factor; GDF-5, growth and differentiation factor 5; IGF-I, insulin-like growth factor I; TGF-βeta, transforming growth factor beta; BMP-12, bone morphogenetic protein 12; PRDX5, peroxiredoxin; CXCL13, CXC chemokine ligand 13; SCX, scleraxis; MKX, Mohawk; shRNA TOB1, short hairpin RNA against the transducer of ERB2,1; miR-135a ROCK1, microRNA against Rho-associated coiled-coil protein kinase 1

Strategies to manage tendon injuries via gene transfer protocols have thus far been based on the administration of sequences coding for (Fig. 2 and Table 2):

* Matrix molecules (tenomodulin - Tnmd, periostin) (Jiang et al. 2016, Noack et al. 2014).
* Growth factors (platelet-derived growth factor B - PDGF-B, vascular endothelial growth factor - VEGF, basic fibroblast growth factor - FGF-2, growth and differentiation factor 5 - GDF-5, insulin-like grwoth factor I - IGF-I, TGF-βeta, bone morphogenetic protein 12 - BMP-12) (Basile et al. 2008, Cai et al. 2013, Hasslund et al. 2014, Lou et al. 2001, Majewski et al. 2008, 2012, Nakamura et al. 1998, Rickert et al. 2005, Schnabel et al. 2009, Tang et al. 2008, 2014, 2016, Wang et al., 2004, 2005, 2007).
* Anti-inflammatory molecules (peroxiredoxin - PRDX5) (Yuan et al. 2004) and chemokines (CXC chemokine ligand 13 - CXCL13) (Tian et al. 2015).
* Transcription factors (scleraxis - SCX, Mohawk - MKX) (Chen et al. 2014, Otabe et al. 2015).
* Signaling molecules (short hairpin RNA against the transducer of ERB2,1 - shRNA TOB1, microRNA against Rho-associated coiled-coil protein kinase 1 - miR-135a ROCK1) (Chen et al. 2015, Gao et al. 2016).

**Table 2:** Gene therapy applications for tendinopathies

Systems	Genes	Applications	References
Non-viral vectors	Tnmd	tenogenesis in vitro and in vivo	Jiang et al. 2016
	PDGF-B	tendon repair in vitro and in vivo	Nakamura et al. 1998, Wang et al. 2004
	VEGF	tendon repair in vitro	Wang et al., 2005
	PRDX5	tenogenesis in vitro	Yuan et al. 2004
	CXCL13	tendon-bone healing in vivo	Tian et al. 2015
	SCX	tenogenesis in vitro, tendon repair in vivo	Chen et al. 2014
AdV vectors	FGF-2	tenogenesis in vitro	Cai et al. 2013
	GDF-5	tendon repair in vivo	Rickert et al. 2005
	IGF-I	tendon repair in vivo	Schnabel et al. 2009
	TGF-β	tendon repair in vivo	Majewski et al. 2012
	BMP-12	tenogenesis in vitro, tendon repair in vivo	Lou et al. 2001, Majewski et al. 2008
	MKX	tenogenesis in vitro, tendon repair in vivo	Otabe et al. 2015
RV/LV vectors	Periostin	tenogenesis in vitro, tendon repair in vivo	Noack et al. 2014
	shRNA TOB1	tendon-bone healing in vivo	Gao et al. 2016
	miR-135a ROCK1	tenogenesis in vitro	Chen et al. 2015
rAAV vectors	VEGF	tendon repair in vivo	Tang et al. 2016
	FGF-2	tenogenesis in vitro, tendon repair in vivo	Tang et al. 2008, 2014, 2016, Wang et al., 2005, 2007
	GDF-5	tendon reconstruction in vivo	Basile et al. 2008, Hasslund et al. 2014

*AdV* adenoviruses, *RV* retroviruses, *LV* lentiviruses, *rAAV* recombinant adeno-associated virus vectors, *Tnmd* tenomodulin, *PDGF-B* platelet-derived growth factor B, *VEGF* vascular endothelial growth factor, *PRDX5* peroxiredoxin, *CXCL13* CXC chemokine ligand 13, *SCX* scleraxis, *FGF-2* basic fibroblast growth factor, *GDF-5* growth and differentiation factor 5, *IGF-I* insulin-like growth factor I, *TGF-β* transforming growth factor beta, *BMP-12* bone morphogenetic protein 12, *MKX* Mohawk, *shRNA TOB1* short hairpin RNA against the transducer of ERB2,1, *miR-135a ROCK1* microRNA against Rho-associated coiled-coil protein kinase 1

The following experimental approaches have been developed to achieve these goals:

* Gene transfer in vitro in differentiated tenocytes and progenitor cells to promote cell survival and tenogenesis with expression of collagen (I/III) markers (Tnmd, periostin, PDGF-B, VEGF, FGF-2, GDF-5, PRDX5, CXCL13, SCX, MKX, shRNA TOB1, miR-135a ROCK1) (Cai et al. 2013, Chen et al. 2014, 2015, Gao et al. 2016, Jiang et al. 2016, Noack et al. 2014, Otabe et al. 2015, Rickert et al. 2005, Tian et al. 2015, Wang et al. 2004, 2005, 2007, Yuan et al. 2004).
* Gene transfer in vivo in various animal models, promoting neotendon formation and tendon healing (Tnmd, periostin, PDGF-B, VEGF, FGF-2, GDF-5, IGF-I, TGF-β, BMP-12, CXCL13, SCX, MKX, shRNA TOB1) (Basile et al. 2008, Chen et al. 2014, Gao et al. 2016, Hasslund et al. 2014, Jiang et al. 2016, Lou et al. 2001, Majewski et al. 2008, 2012, Nakamura et al. 1998, Noack et al. 2014, Otabe et al. 2015, Rickert et al. 2005, Schnabel et al. 2009, Tang et al. 2008, 2014, 2016, Tian et al. 2015).

In summary, gene therapy is an attractive strategy for tendinopathies by providing candidate sequences that mediate neotendon formation and tendon healing over durable periods of time. Studies in adapted preclinical animal models demonstrated the feasibility of applying such gene-based protocols to treat such tendon injuries, providing reasonable hope for translation to the patients soon.

### Biomaterials

Tendon healing and bioengineering-based regeneration can include cytokine modulation, growth factors and PRP administration, biomaterials implantation, gene and cell-based therapies, and tissue engineering strategies (Müller et al. 2015; Bagnaninchi et al. 2007). However, tendon injuries can vary from a tear to chronic tendinopathy, which makes the development of an optimal treatment very difficult either from clinical and engineering/biological point of views. Biomaterial technological platforms offer the opportunity to address tendon injuries in a unique manner as they can be made to resemble the natural tendon extracellular matrix (ECM). Biomaterials of a natural and synthetic origin have been used for the treatment of clinical syndromes affecting tendons in substitutive, healing and regenerative approaches (Liu and Cao 2015). To successfully achieve their function, biomaterials should be able to form strong and stable fibres and must integrate with the surrounding tissue when implanted in the body. In addition, the biomaterials need to have an adequate architecture and demonstrate good biomechanical performance. In addition, they must be biocompatible, biomimetic, bioresorbable/biodegradable, while presenting low antigenicity.

Natural-based polymers originate from natural sources and are being evaluated in tendon regeneration due to their high availability and low cost. These include silk fibroin (Yao et al. 2016) collagen (Purcel, 2016), gelatin (Selle et al. 2015), and hyaluronan (Liang et al. 2014). They have shown promising results in vitro and in vivo. Decellularized matrices have also been developed for Achilles tendon repair. The clinical outcomes observed with acellular human dermal matrix (AHDM) suggest that it is biocompatible, supports revascularization and repopulation with non-inflammatory host cells and is well integrated by the surrounding tendon tissue at 6 months *post*-implantation (Liden and Simmons 2009).

Synthetic polymers such as poly lactic acid (PLA), poly caprolactone (PCL) (Banik et al. 2016), and poly urethane (PU) (Evrova et al. 2016) have also been proposed due to their tailorability, reproducibility and the low immunogenicity risk they present.

The formulation of biomaterials is also being attempted in order to improve the mechanical properties of biomaterials. Cellulose nanocrystals were used to reinforce natural/synthetic polymer blend matrices of poly-ε-caprolactone/chitosan (PCL/CHT) (Domingues et al. 2016).

Similarly, PLA based copolymers blended with collagen and chondroitine sulfate showed good tissue integration and have made for neotissue synthesis after 12 weeks of subcutaneous implantation in rats. Those outcomes provide encouraging results that suggest them being used as scaffolds for tendon and ligament regeneration (Pinese et al. 2017).

The processing of biomaterials as scaffolds has been extensively and interestingly reviewed by others (Francois et al. 2015; Lomas et al. 2015). Moreover, the processing of scaffolds with a multiscale structure and hierarchical organisation has attracted a great deal of interest as it mimics best native tissue organisation and properties (Domingues et al. 2016; Kew et al. 2011).

A study by Wang et al. reported the development of a composite tendon scaffold with a continuous and heterogeneous transition region mimicking a native ligament insertion site (Wang et al. 2015). Decellularized rabbits’ Achilles tendons were used in combination with cells that have been genetically modified to fabricate a stratified scaffold containing three biofunctional regions supporting fibrogenesis, chondrogenesis, and osteogenesis. The in-vitro study showed that a transitional interface could be replicated on the bioengineered tendon.

Silk fibroin has been processed as textiles and cord by using knitting and twisting methods for utilization in tissue engineering of anterior cruciate ligaments (Woods and Holland 2015; Altman et al. 2002).

Electrospinning is a low cost technique that has been explored for tendon nanoscaffolding development (Velasco et al. 2016). That technique is very versatile and allows envisioning the encapsulation of relevant growth factors (e.g. PDGF-BB) from an electrospun polyesther urethane scaffold for tendon rupture repair (Evrova et al. 2016).

3D bio-printing is starting to emerge as a advanced technique that addresses the great challenges in tissue interface regeneration. Merceron and coworkers (Merceron et al. 2015) reported on thermoplastic polyurethane (PU) co-printed on one side with a cell-laden hydrogel-based bioink for muscle development, and poly(−caprolactone) (PCL) co-printed on the other with cell-laden hydrogel-based bioink for tendon development. An in vitro study demonstrated the versatility of this dual system for adequately addressing the challenges of muscle-tendon tissue engineering. 3D bio-printing technology has been used as a possible strategy to generate customizable fiber arrays and reinforce the strength of scaffolds (Mozdzen et al. 2016).

MRI is an accurate technique used in the evaluation of tendinopathies (Yablon and Jacobson 2015) By combining MRI data with reverse engineering, we can look forward to boosting the performance of biomaterials from the architectural and anatomical points of view. The commercial exploitation of such patient-specific implants,that can respect patient anatomy is still an undetermined but their effective production will contribute to improving clinical outcomes and patient quality of life.

### Surgical approach

Based on recent research using immune-histochemical analysis of tissue biopsies from patients with midportion and insertional Achilles tendinopathy and proximal patellar tendinopathy, new non-tendon-invasive treatment methods have been invented. These methods have shown good clinical results, few complications and decreased tendon thickness together with improved tendon structure over time. Surgical treatment should be considered when more conservative treatments fail.

#### Ultrasound and Doppler guided mini surgical scraping and plantaris tendon removal for chronic painful midportion Achilles tendinopathy

Using Ultrasound (US) and color Doppler power (CD), a localized high blood flow was found outside and inside (in close relation to regions with structural changes) the ventral side of the tendon in midportion tendinopathy tendons, but not in normal Achilles tendons (Ohberg et al. 2001). Immune-histochemical analysis of tissue specimens, taken with US and CD-guidance, outside and inside the region with tendon changes showed multiple sympathetic but also sensory nerves outside (ventral side of the tendon). Despite that, there are very few nerves inside the Achilles tendon midportion (Andersson et al. 2007). The nerves were found in close relation to blood vessels. The production of pain substances in tenocytes has been demonstrated (Andersson et al. 2008; Bjur et al. 2008) and a theory about a possible cordless communication between the production of pain substances inside the tendon and nerves outside the tendon has been introduced.

Two studies found amount of type I collagen decreased, increase of type III collagen and GAG. The fibroblasts are remodelled to so-called “myofibroblasts” with (now) contractile properties and there are also small nerves into the tendon, similar to neo-vascularisation (van Sterkenburg and van Dijk CN 2011; Järvinen et al. 1997). Ultrasound and Doppler-guided injections of small volumes of a local anesthetic, targeting the region with high blood flow (blood vessels and nerves) outside the ventral tendon, temporarily cured the pain (Alfredson et al. 2003). These findings led to the invention of a new treatment approach targeting the region with high blood flow and nerves outside the ventral tendon. First, sclerosing polidocanol injections were used and showed promising clinical results in scientific studies (Alfredson and Ohberg, 2005a, b). However, this injection treatment was operator dependent (a long learning curve), multiple injection treatments were often needed and results were not predictable. Therefore, a one-stage mini surgical approach, US and CD-guided mini-surgical scraping, was invented.

Over the last 6 years of research, knowledge has been growing about the plantaris tendon and its possible involvement in midportion Achilles tendinopathy (Alfredson, 2011a, b; van Sterkenburg et al. 2011; Spang et al. 2013; Masci et al. 2015). The role of the plantaris tendon in midportion Achilles tendinopathy is still unclear, but it is tempting to believe that the plantaris is involved in at least a subgroup of patients, especially the ones where the plantaris is located close to the Achilles, sometimes even invaginated into the medial side of the Achilles or inserting into the Achilles (Masci et al. 2015; Alfredson, 2011a, b). In a recent Thesis by Spang, it was shown that the plantaris tendons showed tendinosis features (Spang et al. 2013) and that the connective tissues between the plantaris and Achilles were richly innervated (Spang et al. 2015). Furthermore, 2/3 of all excised plantaris tendons were innervated with sensory nerves. This contrasts with the Achilles midportion where there are few nerves. For patients with tendinopathy and medial side pain, these new findings strengthen the indication to remove the plantaris tendon together with the connective tissue in between the tendons, thereby removing possible compressive forces and nerve rich tissues. The newly invented surgical procedure is presented below.

#### Ultrasound and Doppler-guided mini surgical scraping and plantaris tendon removal

In all patients, after painful tendon loading activity, the clinical diagnosis was confirmed with ultrasound (US) and Colour Doppler (CD) examination that evidenced a thickened Achilles midportion with irregular tendon structure and locally high blood flow outside and inside the regions with structural tendon changes on the ventral (deep) side of the Achilles. The surgical procedure was guided by the US+CD findings.

##### Surgical procedure

**Fig. 3:**
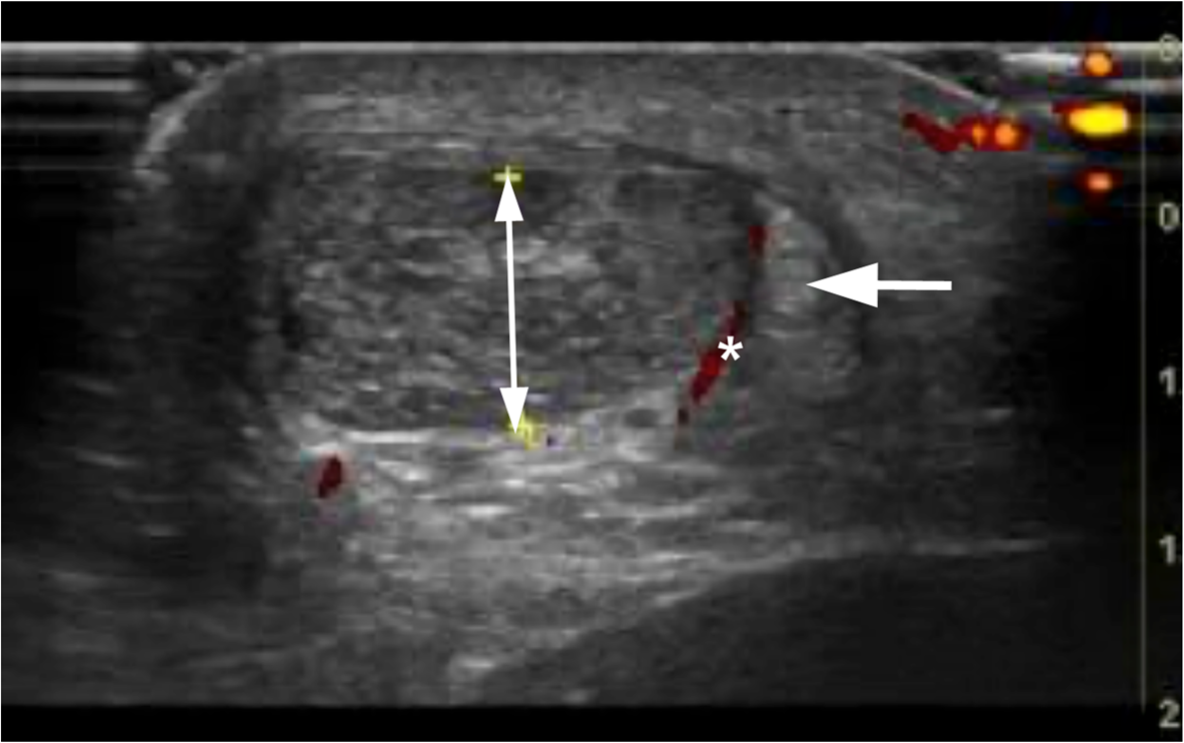
US+CD picture showing plantaris tendon (arrow) placed close to the medial side of a thickened Achilles midportion (doble head-arrow) with high blood flow (*) in between the tendons

After washing, 5-10 ml of a local anesthetic (Xylocain+Adrenaline, 5 mg/ml) was injected on the medial and ventral side of the Achilles midportion. The skin was draped with a sterile paper-cover, exposing only the midportion of the Achilles tendon. A longitudinal skin incision (1–1,5 cm) was made on the medial side of the Achilles midportion, and the Achilles tendon was carefully identified. If the plantaris tendon was found to be positioned close to the medial side of the Achilles, it was carefully released (Fig. 3). The plantaris was followed proximally and cut slightly above the level for the lower medial soleus insertion, followed distally and cut as close as possible to the distal insertion. Most often, 5 to 8 cm of the plantaris tendon was removed. There was often richly vascularized fatty tissue interposed between the Achilles and the plantaris tendon. After removing the plantaris tendon and the fatty tissue between the plantaris and Achilles tendon, the traditional scraping procedure was performed (Alfredson 2011a, b). Outside (ventral side) the regions with structural tendon changes (US) where the CD showed high blood flow, the tendon was completely released from the ventral soft tissue (staying close to the ventral side of the tendon) by dissection with a scalpel. This was followed by hemostasis using diatermia. The skin was closed with single non-resorbable sutures.

#### Postoperative rehabilitation

- Day 1 (Surgery day): Rest, elevated foot.
- Day 2: ROM (Range of movement), light stretching, and short walks.
- Day 3–7: Gradually increased walking activity.
- Day 8–14: Light bicycling.
- After 2 weeks: Sutures out, gradually increased load up to free activity.

Follow-up studies on patients treated with this method have been presented (Ruergard and Alfredson 2014) that show a high success rate and few complications. There are on-going studies following up with larger materials at different activity levels.

#### Ultrasound and color Doppler-guided surgery for insertional Achilles tendinopathy

There is more knowledge about the pathogenesis of the painful midportion Achilles tendon than about its insertion. In both conditions, ultrasound (US) with color Doppler (CD) have shown high blood flow in the painful tendons in contrast to the tendons of pain-free individuals (Knobloch et al. 2006).

Apart from the pathological distal Achilles tendon itself, other tissues have been associated with insertional pain. Bursitis in the retrocalcaneal (van Dijk et al. 2011) and subcutaneous bursae, a skeletal prominence located postero-superior at the calcaneal tuberosity (Haglund’s deformity) causing a tendon-calcaneal impingement (Myerson and McGarvey 1998) and the presence of bone formations and calcifications in the Achilles tendon insertion have all been associated with posterior heel pain. The plantaris tendon could also be potentially associated with this condition (Lintz et al. 2011).

When conservative treatment fails, surgery is indicated. Many different surgical techniques have been described such as extirpation of the retrocalcaneal bursa and a resection of the upper calcaneus (Wiegerinck et al. 2013) and detachment of the Achilles tendon at its insertion followed by removal of intra-tendinous bone formations and calcifications. For intra-tendinous bone formations and calcifications, most surgical methods described include tendon invasive procedures and require long postoperative rehabilitation periods. There is no consensus regarding the most efficient surgical treatment for insertional Achilles tendinopathy (Wiegerinck et al. 2013).

Recent results from histochemically examined tissue samples from the subcutaneous and retrocalcaneal bursa, the upper calcaneus, fatty and fibrous tissue ventral to the distal Achilles tendon collected during insertional Achilles tendon surgery show rich innervation patterns, especially in the subcutaneous bursae (Alfredson and Isaksson 2014). These findings have led to the invention of a new treatment approach for patients having a combination of pathology in the subcutaneous (superficial) bursa, the retrocalcaneal bursa, Haglund deformity and distal Achilles Tendinopathy. Patients with bone spurs, bone bridges and loose bone fragments in the insertion are not included. A description of the treatment method follows.

#### Ultrasound and color Doppler-guided surgery for insertional Achilles tendinopathy

A pre-operative high-resolution grey scale US and CD examination with a linear multi-frequency (8-13 MHz) probe was used during the surgery. Examination of the Achilles insertion showed enlarged subcutaneous and retro-calcaneal bursae with high blood flow inside and outside the bursa walls. There was a prominent upper calcaneus (Haglund deformity) and the distal Achilles tendon was thickened with structural tendon changes located in the ventral and central parts of the tendon. There was also high blood flow inside and outside the ventral part of the distal tendon.

##### Surgical procedure

The operation was carried out with the patient under local anesthesia (5-10 ml of Xylocaine 10 mg/ml with adrenaline 5 μg/ml) infiltrated into the subcutaneous tissues, inside and around the superficial and deep bursae, towards the periosteum of the upper calcaneus, and on the ventral side of the distal Achilles tendon. After 10–15 min, the surgical procedure was started. Through a lateral or laterodorsal longitudinal skin incision about 4–6 cm in length, the subcutaneous tissues were visualized. The first step was to locate the subcutaneous bursa between the skin and the insertion of the Achilles tendon. First, the posterior part of the bursa was carefully dissected from the skin. Then the anterior part was separated from the tendon. The whole bursa was removed. The second step was removal of the retrocalcaneal bursa. This bursa is located between the posterior smooth surface of the superior calcaneal tuberosity and the ventral side of the distal Achilles tendon. The bursa was visible by lifting the Achilles tendon posteriorly, and the bursa was then carefully dissected from the ventral tendon and removed. The third step was scraping the ventral side of the distal Achilles tendon. The infiltrative fatty tissue (including the blood vessels and accompanying nerves) outside the ventral Achilles was carefully scraped loose with a scalpel. The fourth step was to remove the prominent upper calcaneus (Haglund’s deformity). This was done by using an osteotome. By placing the index finger between the tendon and upper calcaneus while a dorsiflexion the ankle joint is produced, the remaining impingement was eliminated. Finally, the cavities were flushed with 4 to 5 ml of Marcain and loose bone ossicles were removed. Hemostasis was carefully established. The skin incision was sutured with non-resorbable sutures.

#### Postoperative rehabilitation

- Day 1: Rest with elevated foot.
- Weeks 1–6: Range of motion exercises and partial weight bearing (up to 50% of full body weight) during slow walking the first 2 weeks. Then full weight-bearing loading and gradually increased walking distances at a slow pace. Light bicycling with the pedal centered under the foot starting 4 weeks after the operation.
- 3 weeks: Suture removal.
- Weeks 7–12: Free walking and high-intensity bicycling. Start with balance and coordination exercises, isometric, concentric and eccentric strength training.
- After 12 weeks: Start slow jogging for short distances, mixed with walking (50-m jog followed by 100-m walk etc.) After 16 weeks: Full tendon loading sport activities.

#### Ultrasound and color Doppler-guided arthroscopic shaving for proximal patellar tendinopathy (Jumper’s knee)

**Fig. 4:**
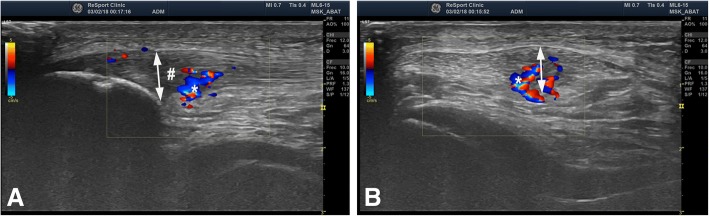
US+Doppler picture (Longitudinal (**a**) and transversal (**b**) view) from a patient suffering from proximal patellar tendinopathy-Jumper’s knee, showing a thickened patellar tendon (doble head-arrow) with structural changes and hypo-echoic regions (#) together with high blood flow (*) inside and outside the dorsal side of the tendon

Proximal patellar Tendinopathy-Jumper’s knee is well-known to be a troublesome to treat (Fig. 4). The conservative treatment of chronic patellar tendon pain-tendinopathy/jumper’s knee using painful eccentric quadriceps training has shown some good results (Purdam et al. 2004). However, this treatment has been less successful among athletes involved in jumping sports. Traditional surgical treatment most often includes open or arthroscopic patellar tenotomy and excision of the region with tendon changes. Sometimes, ultrasound-guided percutaneous longitudinal tenotomy, curettage, multiple drilling of the inferior patellar pole or excision of the distal patellar tip is used (Testa et al. 1999). After these intra-tendinous treatments, there is a relatively long rehabilitation period. The clinical results of these types of intra-tendinous surgeries have been shown to be varying (Coleman et al. 2000). In a randomised study comparing treatment with eccentric quadriceps training and traditional open tenotomy plus excision, there were similar but only 50% good clinical results in the groups (Bahr et al. 2006).

Over recent years, where the pain comes from in this and other chronic painful tendinopathies has been debated (Khan et al. 2000). Recent studies using US+CD and immuno-histochemical analyses of tendon biopsies have shown high blood flow (Alfredson and Ohberg 2005a, b) and nerves (Danielson et al. 2008) outside the tendon (on the dorsal side of the proximal patellar tendon). There were very few, if any, nerves inside the tendons. Local anesthetic injections targeting the region with high blood flow and nerves outside the dorsal side of the tendon temporarily cured the pain. These findings have led to research on new treatment methods like sclerosing polidocanol injections (Hoksrud et al. 2006) and ultrasound-guided arthroscopic shaving (Willberg et al. 2007), focusing the treatment outside the dorsal patellar tendon, i.e. where the high blood flow and nerves have been demonstrated. Below the newly invented surgical treatment method is presented.

##### Surgical procedure

**Fig. 5:**
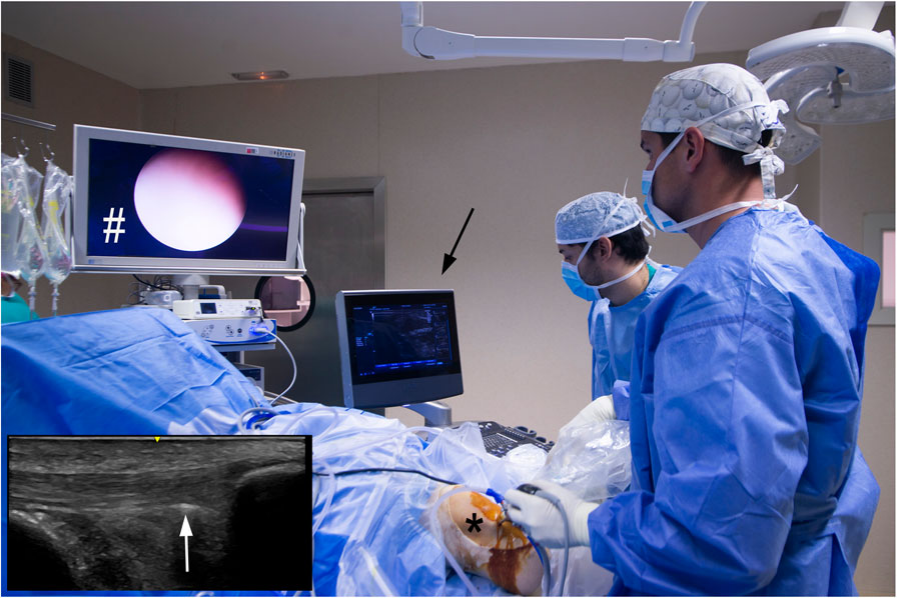
Pictures showing the US-guided (black arrow) arthroscopic (* and #) surgical set-up. Small picture shows the ultrasound view, white arrow pointing at the shaver positioned on the deep side of the proximal patellar tendon

The US and CD examination guides the arthroscopic surgical procedure (US examination together with arthroscopy in the operating room) that aims to be minimally invasive outside (dorsal side) the proximal patellar tendon (Fig. 5).

Arthroscopy is performed under local anaesthesia. The patients are in the supine position with the knee straight and quadriceps relaxed. Standard antero-medial and antero-lateral portals and a pressure controlled pump were used. No tourniquet is used. Initially, a standard arthroscopic evaluation of the whole knee joint is performed. Then, the patellar tendon insertion into the patella is identified. For shaving, a 4.5 mm full radius blade shaver is used. Simultaneous ultrasound examination (longitudinal and transversal views) guides the procedure. Careful shaving, aiming to destroy only the region with high blood flow (neovessels) and nerves adjacent to the tendinosis changes on the dorsal side of the tendon, is performed (i.e. separating the Hoffa fat pad from the patellar tendon). No tendon tissue is resected and the Hoffa fat pad is touched as little as possible. The portals are closed with sutures or tape and a bandage is used for 24 h.

#### Postoperative rehabilitation

The patients are allowed full weight-bearing walking immediately after the treatment. Because no intra-tendinous surgery is performed, the following rehabilitation can start immediately and be relatively aggressive and quick. Range of motion exercises and enhancement training, immediate weight-bearing loading, biking and low-load strength training begin within the first 3 weeks. Then, there is a gradual increase in loading and start of more sport specific training depending on swelling and pain. Isometric, concentric and eccentric exercises should be tolerated before plyometric training is instituted.

The rehabilitation periods needed varies from 2 to 4 months before returning to full tendon loading sports activity.

This new method has been evaluated in several scientific studies and in a doctoral thesis (Sunding et al. 2015). The method has been shown to be safe. The results have been shown to be very good and stable with more than 85% satisfied athletes returning to full sport activity within 3–4 months. Altogether, we have operated more than 700 athletes with this method. They include rugby (Alfredson and Masci 2015), football, volleyball and track and field athletes. The results have been good and stable.

In patients that have previously been treated with tenotomy and revision, the US+DP-guided procedure has been less successful.

Intra-tendinous surgical approaches reported poor clinical results. This fact in combination with the innervation patterns makes this surgical approach questionable. Surgical treatment outside the tendon, such as US and CD-guided arthroscopic shaving, has been shown to have a high potential for allowing for a pain-free return to high-level patellar tendon demanding sports after a relatively short rehabilitation period.

## Conclusions

In the present review different therapeutic options are shown. It is recommended to start with the less invasive ones, moving towards more invasive options if the conservative treatment fails.

## Abbreviations

AHDM: Acellular human dermal matrix
ASCs: Adipose-derived mesenchymal stem cells
BMSCs: Bone marrow stromal cells
CD: Color Doppler
CTGF: Connective tissue growth factor
ECM: Tendon extracellular matrix
EDTA: Ethylene-diaminetetraacetic acid
EGF: Epidermal growth factor
ESSKA: European Society of Sports Traumatology, Knee Surgery and Arthroscopy
HGF: Hepatocyte growth factor
IL: Interleukine
LP-PRP: Leukocyte-poor PRP
LR-PRP: Leukocyte-rich PRP
MMPs: Metalloproteinases
MSCs: Mesenchymal stem cells
NSAIDs: Nonsteroidal anti-inflammatory drugs
PCL: Poly caprolactone
PCL/CHT: Poly-ε-caprolactone/chitosan
PDGF: Platelet-derived growth factor
PLA: Poly lactic acid
PPAR-gamma: Peroxisome proliferation receptor gamma
PRP: Platelet Rich Plasma
PU: Poly urethane
ROM: Range of movement
TGF: Transforming growth factor
TGF-β1: Transforming Growth Factor Beta − 1
US: Using Ultrasound
USGET: UltraSound-guided Galvanic Electrolysis Technique
VEGF: Vascular endothelial growth factor
